# Study protocol for the use of propofol in adult intensive care unit patients: a secondary analysis of an extensive international database

**DOI:** 10.62675/2965-2774.20260176

**Published:** 2026-02-20

**Authors:** Wolfgang H. Hartl, Christian Stoppe, Yuki Kotani, Giovanni Landoni, Andrew G. Day, Johannes Piller, Michael Neuberger, Andreas Bender

**Affiliations:** 1 University Medical Center Ludwig Maximilian University Department of General Munich Germany Department of General, Visceral, and Transplantation Surgery, University Medical Center, Campus Grosshadern, Ludwig Maximilian University - Munich, Germany.; 2 University Hospital Würzburg Emergency and Pain Medicine Department of Anaesthesiology Würzburg Germany Department of Anaesthesiology, Intensive Care, Emergency and Pain Medicine, University Hospital Würzburg - Würzburg, Germany.; 3 Kameda Medical Center Department of Intensive Care Medicine Kamogawa Japan Department of Intensive Care Medicine, Kameda Medical Center - Kamogawa, Japan.; 4 IRCCS San Raffaele Scientific Institute Department of Anesthesia and Intensive Care Milan Italy Department of Anesthesia and Intensive Care, IRCCS San Raffaele Scientific Institute - Milan, Italy.; 5 Kingston Health Sciences Centre - Kingston Clinical Evaluation Research Unit Ontario Canada Clinical Evaluation Research Unit, Kingston Health Sciences Centre - Kingston, Ontario, Canada.; 6 Ludwig Maximilian University Statistical Consulting Unit Department of Statistics Munich Germany Statistical Consulting Unit, StaBLab, Department of Statistics, Ludwig Maximilian University - Munich, Germany.; 7 Ludwig Maximilian University/Technische Universität Munich Center for Machine Learning Munich Germany Munich Center for Machine Learning, Ludwig Maximilian University/Technische Universität - Munich, Germany.

**Keywords:** Intensive care units, Respiration, artificial, Propofol, Energy intake, Soybean oil, Survival

## Abstract

**Background and aims:**

There are conflicting data regarding the use of propofol in the intensive care unit. The present study aims to explore the propofol dose-response relationship in the context of associations with clinical outcomes. Here we present the data collection and analysis procedures.

**Methods and analysis:**

The International Nutrition Survey (www.criticalcarenutrition.com) contains data from 785 medical, surgical, or trauma intensive care units. This survey also includes data on daily mechanical ventilation and propofol use, along with the associated propofol-related energy intake. Of the 21,100 adult patients, we will analyze those who spent at least 48 hours in the intensive care unit. Data collection will include patient characteristics, propofol parameters, use of mechanical ventilation, severity of illness at intensive care unit admission, and time to discharge alive or in-hospital death. In addition to the duration of propofol therapy (days), propofol-associated fat intake will be used as a surrogate for propofol dose. Statistical analyses will use multistate models and piece-wise exponential additive mixed models to examine associations between propofol use (including associated fat intake) and outcomes, while adjusting for numerous confounders and accounting for mechanical ventilation.

**Ethics and dissemination:**

Institutional ethics approval was granted by the Health Sciences Research Ethics Board at Queen's University, Kingston, Ontario (file number 6004791). Informed patient consent was not required due to the nature of this study. Procedures were conducted in accordance with the ethical standards of the institutional or regional committee for human experimentation and the Helsinki Declaration of 1975.

## INTRODUCTION

Recently, controversy has arisen regarding whether propofol has undesirable side effects compared to other sedative drugs.^(1)^ These effects may mainly occur in patients over 65 years of age,^(2)^ but have also been observed in pediatric populations.^(3)^ A meta-analysis by Kotani et al.^(1)^ involving 252 randomized controlled trials and more than 30,000 patients found that the use of propofol is associated with a potential 10% increase in mortality compared with other sedation strategies. This fatal side effect may also be relevant for critically ill patients, for whom there was a 10% relative increase in mortality (15% *versus* 13%), with a Bayesian approach indicating a 75.7% probability of harm.^(4)^

In contrast, other meta-analyses have found that, compared to midazolam, the use of propofol does not worsen the outcome for a heterogeneous population of critically ill adult patients,^(5)^ and may even reduce the length of stay in the intensive care unit (ICU), the duration of mechanical ventilation (MV) and the time to extubation for acutely ill surgical patients.^(6)^ Two recent meta-analyses compared propofol with dexmedetomidine. On the one hand, there was no difference in mortality.^(7,8)^ However, in the meta-analysis by Heybati et al.,^(7)^ for example, mortality was only an endpoint in four trials involving 364 patients. This small sample size may have contributed to the very low certainty of these negative results. Conversely, Heybati et al.^(7)^ found that propofol may increase the duration of MV and lengthen hospital stays for unselected patients, as well as increasing the risk of ICU-associated *delirium* in cardiac surgery patients.

So far, no study has examined the dose-dependent effects of propofol on outcomes in scenarios where the maximum application rate of 4 - 5mg/kg per hour or 95 - 120mg/kg per day has not been exceeded. Furthermore, examinations of the effects of prolonged propofol administration in controlled studies have been limited to a maximum of five days.^(7)^ It is currently unknown whether the critical illness phase (early or late) in which propofol is administered also affects its unwanted effects. There is also little information on the effect of cumulative propofol doses on outcomes. A recent monocentric retrospective observational study of 839 critically ill, mechanically ventilated patients found no association between substantial cumulative propofol doses (> 500mg) and 6-month mortality.^(9)^ However, it should be noted that, without adjustment for confounders, a high cumulative dose of propofol was associated with shorter survival. This association only disappeared after adjustment, which may be related to the relatively small number of patients studied (less than 1,000).

The primary purpose of this study is to investigate the relationship between the duration of propofol therapy and daily/cumulative propofol-associated energy intake and outcomes.

### Research question

The average causal effect of propofol on the risk of in-hospital all-cause death has been demonstrated in previous clinical trials. Associations between propofol doses, timing, and responses, however, are unknown. The present study aims to fill this gap. The primary research question focuses on the association between defined levels of propofol-associated energy intake in the early and late phases following ICU admission and competing risks (time to discharge alive (being a surrogate of morbidity), and in-hospital death, respectively). The secondary research question centers on the association between preceding cumulated or daily propofol energy intake, or the number of days on propofol prior to transitioning from propofol to, e.g., death or being free of propofol the day after transitioning. These factors will be examined in terms of measured variables and transition probabilities, while accounting for bias from confounding by indication or palliative care. The general aim is to provide more evidence on safe propofol dosing and duration.

## METHODS

### Data base

The International Nutrition Survey is a multinational database of critically ill adults treated in 785 international medical, surgical, or trauma ICUs. This database includes, in addition to nutritional and outcome data, information on daily propofol use and associated fatty acid intake and has collected data on more than 20,000 critically ill patients. Details of this database have been presented in a previous publication, and to date, the results of 18 previous studies have been based on various subgroups included in this database. Details of these studies, which addressed various aspects related to medical nutrition therapy, can be found in the electronic database.^(9)^

The core of the present study will be the analysis of a subpopulation of this multinational database. Patients enrolled in this database were followed from the day of ICU admission. Nutritional and clinical parameters (including daily protein intake, lipid intake, use of MV, propofol, and propofol-associated fat energy intake (including soybean oil and purified egg phosphatide) were collected daily from Day 0 (day of ICU admission) until ICU discharge or death, or for a maximum of 12 days for patients still in the ICU. Survival was assessed until hospital discharge (dead or alive) up to 60 days from ICU admission. Only ICUs with at least one included patient were kept in the database.

During the ICU stay, patients received standard medical care (including MV and propofol) at the treating physician's clinical judgement. The principles of sedation, ventilation strategies, including lung protective ventilation, weaning, and prevention/therapy of ventilator-associated pneumonia, followed international guidelines in effect at the time of the data collection.^(10-13)^

### Selection of the study population

#### Inclusion criteria

Patients selected from the database will be aged 18 years or over and have a body mass index (BMI) of over 13kg/m^2^. Other inclusion criteria will be an ICU length-of-stay (LOS) of > 48 hours. The rationale behind this inclusion criterion is that the potentially detrimental effects of propofol on survival are not attributable to immediate or acute reactions, but instead require more than 48 hours of therapy to manifest (e.g., in the event of propofol infusion syndrome).^(1,14)^

For patients admitted to the same unit during the same hospital stay, we will only analyze the first ICU admission. Patients readmitted after hospital discharge will be included as separate cases.

#### Data collection

Using a secure web-based data collection tool, the following information was collected: date of ICU admission, admission category (elective surgery, emergency surgery, and medical), primary admission diagnosis (nine categories), sex, age, BMI, daily use of MV/propofol therapy, and Apache II score on the day of admission. Attending physicians or allied healthcare professionals with expertise in nutrition practices (e.g., dietitians) also recorded daily protein intake and route of additional caloric intake (enteral nutrition, parenteral nutrition). For the first 11 days after ICU admission, the database contains information on protein and energy intake, as well as the number of days on which a patient received parenteral nutrition or oral feeding at any time.

Daily propofol-associated energy intake was also collected from the day of ICU admission (partial day) to a maximum of 11 additional days after admission. Information on the exact dose of propofol (administered via 1% or 2% solutions) was not available in the database. However, as propofol-associated energy intake is closely related to the dose of propofol, propofol-associated energy intake can be used as a surrogate parameter for this dose (1mL of a solution containing propofol contains 50mg of soybean oil, and 50mg of purified egg phosphatide, equivalent to 1.1kcal/mL).

Patients were followed in the hospital for a maximum of 60 days after ICU admission. We recorded the time to in-hospital death or discharge alive. Patients who remained alive in the hospital for more than 60 days were considered censored for both risks at that time.

#### Sample size considerations

The large number of patients in the database (more than 18,000 with an ICU LOS > 2 days) will be sufficient to support advanced, in-depth statistical analyses. In this subgroup, the 60-day hospital discharge alive rate is approximately 70%, resulting in more than 10,000 events (discharges).^(15)^ Considering the number of planned predictor parameters (confounders, see below), the events per predictor parameter ratio will be large enough to perform reliable complex statistical analyses.^(16)^

### Exposure variables

**Figure 1:**
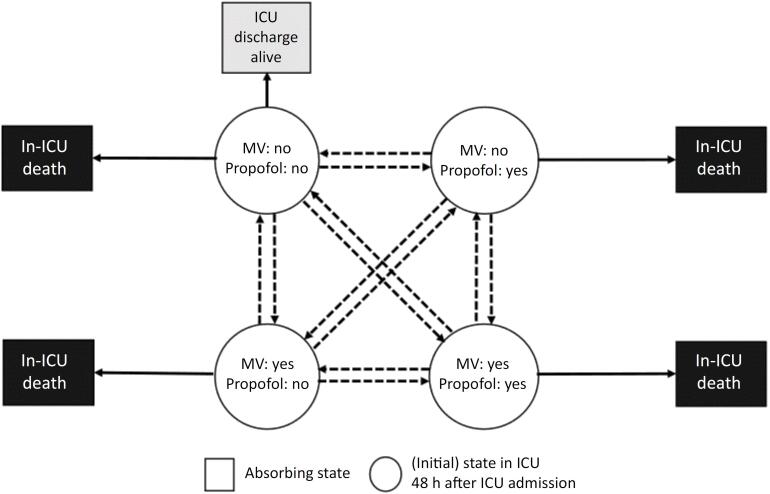
Multistate model describing the transitions between different clinical states.

– Number of days with propofol therapy preceding transition day from one clinical state to another (numeric variable), irrespective of whether a patient had propofol on transition day or not (for definition of state transitions see Figure 1)
– The cumulated amount of propofol-associated energy intake (kcal/kg) (numeric variable) preceding transition day, irrespective of whether a patient had propofol on transition day or not
– The amount of daily propofol-associated energy intake (kcal/kg and day) on transition day (numeric variable)
– The amount of daily propofol-associated energy intake (kcal/kg and day) (categorized variable) during different phases after ICU admission. The associations of daily propofol-associated energy intake with the outcome will be cumulated for the early phase (Days 0 - 4 after ICU admission) and the late phase (Days 5 - 11 after ICU admission).

ICU - intensive care unit; MV - mechanical ventilation.

The initial state on intensive care unit can be any combination of mechanical ventilation (yes/no) and propofol (yes/no). Initial and absorbing states and transitions are depicted by circles and dashed lines respectively. The transition to absorbing state death is of particular interest, particularly how the covariate adjusted transition rates differ between the two mechanical ventilation groups (yes/no), depending on administration of propofol (yes/no). All associations will additionally be adjusted for the number of days a patient had been on mechanical ventilation before transition. Risks for state transitions will be referred separately to a) the number of days a patient has received propofol before transition, or b) the cumulated amount of propofol-associated energy intake (kcal/kg) before transition, or c) the amount of propofol-associated energy intake (kcal/kg and day) on transition day.

### Confounder variables

The list of confounder variables is presented in table 1. To account for heterogeneity across ICUs, all models will include random effects for each unit.

**Table 1:** List of confounder variables. In addition to these variables, all models will contain a random intensive care unit variable

Variable	Time of recording
Number of days on oral nutrition	Days 0 - 2 post ICU admission
Number of days on parenteral nutrition	Days 0 - 2 post ICU admission
Number of days with protein intake > 1.2g/kg day	Days 0 - 2 post ICU admission
Year of therapy	Admission date
APACHE II score	ICU admission
Main admission diagnosis*	ICU admission
Admission category (surgical elective/emergency, medical)	ICU admission
Age	ICU admission
Sex	ICU admission
BMI	Pre-ICU admission
Baseline number of days under MV†	Days 0 - 2 post ICU admission
Number of days under MV before state transition‡	Days 0 - 11 post ICU admission

ICU – intensive care unit; APACHE - Acute Physiology and Chronic Health Evaluation; BMI - body mass index; MV - mechanical ventilation.

* Gastrointestinal, cardio-vascular, metabolic, neurologic, orthopedic/ trauma, renal, pulmonary, sepsis, other. Sepsis was the main admission diagnosis, if there was an infection-associated circulatory dysfunction requiring vasopressor therapy which dominated dysfunction of other organs;

† only used when analyzing the association of daily propofol-associated energy intake (categorized variable) with time-to-discharge alive/in-hospital death (cumulated for the early and late phase after intensive care unit admission);

‡ only used in multistate models

### Outcome measures

– **Primary outcomes:** time-to-hospital discharge-alive as a surrogate of morbidity, and to in-hospital death (adjusted hazard ratios and 95% confidence intervals [95%CI]).
  Adjusted hazard ratios compare two levels of daily propofol-associated energy intake. They will be calculated separately for each day after ICU admission using sub-distributional and cause-specific hazard models. The observation period will start on the day of ICU admission, and events (day of discharge alive/in-hospital death) will be registered from day 2 after ICU admission until the end of the lead-time (defined as twice the number of days that a patient received propofol therapy).
– **Secondary outcomes:** the risk of state transition (adjusted hazard ratio and 95%CI) and the transition probabilities between the different states (Figure 1).
  State transition risks and probabilities will be referred to a certain amount of propofol-associated energy intake or a specific duration of propofol therapy; adjusted hazard ratios will be calculated for all 17 transitions (Figure 1). State transition will be registered until day 13 after ICU admission. Initial and absorbing states include "propofol: no/MV: no", "propofol: yes/MV: no", "propofol: no/MV: yes", "propofol: yes/MV: yes", post-transition absorbing states include "in-ICU death" and "ICU discharge alive.

### Data management plan

All data collected during the study were recorded by designated site personnel in a dedicated repository and stored in a study-specific database.^(17)^ Data from this database were transferred to an electronic data management system that complies with the guidelines for Good Automated Manufacturing Practice, version 5.0 (GAMP 5; INES, IQVIA, Paris, France). All patient data are pseudonymized and coded with a unique patient identification (ID) number generated by the electronic data management system. The database complies with all applicable national, regional, and local regulations on patient data protection and record keeping. Data analysis will be performed using R statistical software. Details of the study will be reported in accordance with the Strengthening the Reporting of Observational Studies in Epidemiology (STROBE) guidelines.

### Statistical analysis plan

#### Descriptive analysis

Continuous variables are described by mean, SD, median, minimum, and maximum values. In addition, two-sided 95%CIs are calculated for continuous variables. Additional descriptive statistics may be calculated as needed (e.g., quartiles, coefficient of variation, etc.). In particular, the median and interquartile range (IQR) are used to describe non-normally distributed data. Frequencies and corresponding percentages per class level describe categorical variables. Patients with missing data will be excluded. However, we will examine where and how frequently missing values occur in the data overall, how often they co-occur across variables, and whether there are notable patterns of missingness across groups. We will then derive possible limitations of this study from that analysis.

For the competing risk analyses (time-to-discharge alive, time to in-hospital death), we will classify daily propofol-associated energy intake using three thresholds that define three different levels based on the amount of propofol-associated energy received. The identification of specific energy thresholds will be based on clinical practice:^(18,19)^ level I: < 1.0kcal propofol-associated energy/kg per day; level II: 1.5 - 4.5kcal propofol-associated energy/kg per day; level III: > 4.5kcal propofol-associated energy/kg per day). Suppose there is a highly uneven distribution of patient days with a given level of propofol-associated energy intake. In that case, energy thresholds may be redefined after preliminary exploratory analysis of these data, but before assessing the association with outcome.

For converting propofol-associated energy intake into mg propofol, we will assume that the use of 1% and 2% propofol in the present study was similar to that in the multinational EuroPN study on medical nutrition therapy (2% propofol: 62%, 1% propofol: 37%, unknown: 1%; unpublished observations of the EuroPN study).^(20)^ From these data, it is possible to convert propofol-associated energy intake into mg propofol. According to Appendix A, the energy levels above would roughly correspond to the following propofol dose levels: level I: < 20mg/kg per day; level II: 20 - 60mg/kg per day; level III: > 60mg/kg per day. These propofol categories, however, will only be used to interpret results. For all statistical analyses, we will use the levels defined above based on propofol-associated energy intake.

### Overall design of the multistate models

We will use multistate models to assess the association between cumulated daily propofol-associated energy intake preceding state transition; propofol-associated energy intake on transition day, and number of days with propofol therapy preceding state transition, adjusted for the use of MV, and the subsequent risk for an inevitable state transition (Figure 1).^(21,22)^ We will analyze state transitions on a daily basis (days 0 to 11 after ICU admission), since the database contains information on propofol use and total propofol-associated energy intake on a given day. There are no data on state transitions occurring within 24 hours. Furthermore, transitions between propofol states have only been recorded up to 12 days after ICU admission. This is because propofol data recording stopped if a patient died or was discharged from the ICU, or if they had been in the ICU for more than 11 days. In line with this, for the multistate models, we will only consider outcomes (absorbing states: in-ICU death and discharge from the ICU alive) up to day 12 after ICU admission.

All analyses (multistate models and competing risks, see below) will be performed using the Piecewise Exponential Additive Mixed Model (PAMM). Like the Cox model, this model class is a multiplicative hazards model that estimates the hazard rate (or, more generally, the transition hazards) conditional on the covariates. Coefficient estimates can be interpreted in the same way as in the Cox model, e.g., using hazard ratios. We will use the PAMM rather than the Cox model because it explicitly estimates the baseline hazard and facilitates the estimation of nonlinear and time-varying effects, as well as the inclusion of time-dependent covariates (e.g., the number of previous days on MV). The general idea is to partition the observation period into short time intervals and estimate a constant hazard rate for each interval. This enables the model to capture varying hazard rates at different times after ICU admission, making it suitable for situations where the risk of state transition changes over time (e.g., after ICU admission). In the context of a multistate model, the hazard by piecewise exponential models becomes the transition hazard. Details can be found in the references.^(9,15,23-26)^

Hazard ratios can be used to describe the association of covariates and exposures with each transition (e.g., from alive to death in the ICU). In multistate settings, separate hazards are estimated for each possible transition. These individual transition hazards are then combined to obtain the overall transition probabilities by calculating the empirical transition matrix. While hazard ratios describe the direct effect on the instantaneous risk of a transition (relative risk), transition probabilities depend on the hazards of all possible transitions (absolute risk). Together, these two quantities provide a comprehensive interpretation of the underlying processes.

A particular focus will be on the concomitant use of MV, which is one of the main reasons for propofol use in critically ill patients and therefore the most important confounder of propofol-associated outcomes. The time-dependent confounder MV is considered as a separate state^(27)^ in the multistate model (Figure 1). This has the advantage that the transitions to the absorbing states "ICU discharge alive" and "in-ICU-death", and thus the transition probabilities to these states, can be modelled separately, depending on the current time-dependent propofol status (yes or no), but also on the subject-specific history of MV treatment and propofol administration (number of days with propofol therapy, cumulated propofol-associated energy intake). As the model is conditional on propofol status at any given time, but the frequency of MV use may vary between subjects within each propofol state, it allows the association of propofol and MV status with outcomes to be disentangled.

Results for the association between the exposure variables preceding transition (number of days with propofol therapy, cumulated propofol-associated energy intake (kcal/kg), propofol-associated energy intake (kcal/kg day) on transition day), and transition risk will be presented as adjusted hazard ratios (with 95%CIs) and adjusted transition probabilities (with 95%CIs) depending on a specific day after ICU admission (Days 1 to 11).

### Overall design of the competing risk models used to analyze time to discharge alive and to in-hospital death

The primary aim will be to analyze the association between daily propofol-associated energy intake from day 1 to day 11 after ICU admission and the time to the hospital endpoint (discharge alive with in-hospital death as a competing risk).

Recording of propofol-associated energy intake was stopped if a patient died, was discharged from the ICU, or was in the ICU for more than 11 days. However, our statistical model requires information on propofol-associated energy intake on propofol days 1 to 11 after ICU admission for all surviving patients, even if patients were discharged from the ICU before day 11. Assuming no propofol-associated energy intake after ICU discharge alive, we will impute a daily propofol-associated energy intake of 0 for these patients.

To account for the fact that a short duration of propofol therapy (and propofol-associated energy intake) may not affect outcome over a more extended period, we will use a lead-time defined as twice the number of days that a patient received propofol therapy. These lead times provide a window in which propofol-associated energy intake on a given day could have affected the subsequent day of discharge alive (time to discharge alive).

To facilitate the interpretation of the associations between propofol-associated energy intake and outcome estimated by our model, we will construct five different hypothetical propofol-associated energy intake use patterns (reflecting three different levels of daily propofol-associated energy intake (low, medium, high) on propofol days 1 to 11). These levels of use will also be used to model associations between propofol-associated energy intake and outcomes. Similar to our recent publications,^(9,15)^ we will distinguish between an early (days on propofol #0 to #4) and a late (days on propofol #5 to #11) acute phase.

We will then use these models to compare the expected outcomes of hypothetical propofol-associated energy intake patterns, controlling for confounders. We will design six different pairwise comparisons of these artificial patterns, analyzing the events of interest (separate models for early acute phase, late acute phase, or early + late acute phase: comparison of intermediate propofol-associated energy intake *versus* low propofol-associated energy intake, and high propofol-associated energy intake *versus* intermediate propofol-associated energy intake). All adjusted hazard ratios (pairwise comparisons of different hypothetical propofol-associated energy intake patterns) will be calculated assuming ceteris paribus.

The primary objective of the analysis of propofol-associated energy intake is to obtain the cumulative incidence function of the events of interest (time-to-discharge alive, taking into account the competing event of in-hospital death). We will again use PAMM. We will use techniques employed in previous publications^(9,15)^ to model cumulative effects where past values of a propofol-associated energy intake influence the hazard on a given day after ICU admission. This is relevant when accumulated propofol exposure may increase the risk of an event.

To model the cumulative effects over the follow-up period, we will use the lead time described above. This describes the time interval during which a propofol-associated energy intake registered on a given day after ICU admission affects the likelihood of hospital discharge alive or in-hospital death. We will first create the piecewise exponential data format by constructing covariate matrices for propofol-associated energy intake, additional matrices representing the time of exposure, and a lead matrix defining the time window during which the event of interest (hospital discharge alive or in-hospital death) will be recorded. Furthermore, we will divide the time axis after ICU admission into segments (Day x to Day x + 1 after ICU admission), assuming a constant exponential distribution within each segment.

Propofol-associated energy intake is a time-dependent covariate characterized by time-varying exposure. This time-varying exposure can be described by an individual's exposure history, in which a specific value of propofol-associated energy intake is attributed to each day after ICU admission. To model the cumulative associations, we will first compute the partial association over the time window containing the aforementioned segments, in which the value of propofol-associated energy intake on a given day after ICU admission can affect the hazard rates. For cumulation, we will use the weighted cumulative exposure model,^(28)^ in which the partial associations are assumed to be linear and depend only on the time window. This model is defined as the definite integral of the partial associations over the entire time window. Furthermore, the model computes the cumulative associations between time-varying propofol-associated energy intake and recency.

For the primary analysis, we will first fit cause-specific hazard models for each event type, and then combine the resulting models to estimate the cumulative incidence function for each event type.^(15)^ The cumulative incidence function is calculated by considering the probability of hospital discharge alive or in-hospital death until the end of the lead time. For comparison, we will also use the Fine–Gray subdistribution hazard model to analyze predictors (including the cumulative association of propofol-related energy intake) of time-to-discharge alive.^(29)^

The Fine-Gray model is a type of proportional hazards model used to estimate the probability of the event of interest (hospital discharge alive) occurring, while accounting for in-hospital death (a competing risk), which can prevent a patient from being discharged alive from the hospital. The Fine-Gray model directly estimates the cumulative incidence function of the cause of interest (discharge alive), and the advantage of this model is that the effects of covariates and exposures can be interpreted directly with respect to the cumulative incidence function. The general estimation approach will be equivalent to the cause-specific approach described above; however, there will be no need to model the competing event directly.

For time to discharge alive/in-hospital death, we will present adjusted hazard ratios (with 95%CIs) comparing two levels of cumulative propofol-associated energy intake. Adjusted hazard ratios will be calculated separately for each day after ICU admission in the defined time window. Cumulative incidence functions will show the estimated proportion (with 95%CIs) of patients who have been discharged from the hospital alive on a given day after ICU admission, by their cumulative propofol-associated energy intake.

### Subgroup analysis

In order to look at associations according to admission diagnosis, sex and age, the results will be analyzed separately for female patients, surgical patients with a cardio-vascular admission diagnosis, patients who only had a low propofol-associated energy intake during the first 48 hours after ICU admission (daily propofol-associated energy intake (level I): < 1.5kcal/kg per day), patients younger than 65 years and patients with an admission APACHE II score ≥ 25.

### Sensitivity analyses accounting for palliative care bias/confounding by indication

To account for palliative care bias in the multistate models and analysis of time-to-discharge alive/in-hospital death, a sensitivity analysis is required concerning patients with an early death after termination or beginning of propofol therapy (< 48 hours after the last day of propofol therapy). Palliative care bias arises if physicians modify therapy not for medical reasons but because of end-of-life decisions. Several scenarios are possible (Table 2):

**Table 2:** Survey of sensitivity analyses that account for bias relating to indications and palliative care

	Primary outcome	Secondary outcome		
Outcome measures	Time-to-discharge-alive/in-hospital death	Hazard of state transition		
Exposure variables	Categorized daily propofol-associated energy intake (kcal/kg and day)	Number of days with propofol therapy preceding transition day	Cumulated amount of PAEI (kcal/kg) before state transition (initial state: propofol "yes")	Amount of daily PAEI (kcal/kg and day) on transition day (initial state: propofol "yes")
Sensitivity analyses	Use of a lag time of two days after the last day of propofol therapy	Re-definition of the absorbing state "in-ICU death" into "palliative in-ICU death" (for time courses of different daily states #1 to #9, Table 3) and "non-palliative in-ICU death" (all other state time series)	Re-definition of the absorbing state "in-ICU death" into "palliative in-ICU death" (if [PAEI on transition day minus PAEI on the day before transition day]/PAEI on the day before transition day ≥ 100%) and "non-palliative in-ICU death" (all other changes)	Re-definition of the absorbing state "in-ICU death" into "palliative in-ICU death" (if PAEI on transition day > 4.5kcal/kg day) and "non-palliative in-ICU death" (all other intake)

PAEI - propofol-associated energy intake; ICU - intensive care unit.

### Sensitivity analysis for the multistate models

If it is decided to withdraw established life-sustaining therapies (e.g., MV) based on the presumed wishes of the patient (e.g., communicated by a surrogate) or the instructions of an advance directive, sedation may be changed to drugs stronger than propofol (midazolam), and opioids (morphine, etc.) are added to analgo-sedate the patient. Death usually occurs one to two days after these therapeutic changes. Consequently, in these cases, the transition from propofol to no propofol would not indicate an improvement in clinical status (as it would otherwise). To account for this bias within the transition from the state "propofol: yes" to the state "propofol: no", we will re-classify the subsequent absorbing state "in-ICU death" into "palliative in-ICU death", if death occurs one or two days after the preceding state transition (time course of states # 1 - 7, Table 3), and into "non-palliative death" in all other time courses of states.

**Table 3:** Survey of time courses of different daily states used to re-define the absorbing state "in-intensive care unit death" into "palliative in-intensive care unit death" (time course of states #1 to #9) and "non-palliative in-intensive care unit death" (all other time courses)

Time course of states #	State on day x minus n (n > 2)	State on day x minus 2	State on day x minus 1	Initial state (day x)	Absorbing state (day x plus 1)
1	Any	Any	"Propofol: yes" "MV: yes"	"Propofol: no" "MV: yes"	In-ICU death
2	Any	Any	"Propofol: yes" "MV: no"	"Propofol: no" "MV: no"	In-ICU death
3	Any	Any	"Propofol: yes" "MV: yes"	"Propofol: no" "MV: no"	In-ICU death
4	Any	"Propofol: yes" "MV: yes"	"Propofol: no" "MV: yes"	"Propofol: no" "MV: yes"	In-ICU death
5	Any	"Propofol: yes" "MV: no"	"Propofol: no" "MV: no"	"Propofol: no" "MV: no"	In-ICU death
6	Any	"Propofol: yes" "MV: yes"	"Propofol: no" "MV: yes"	"Propofol: no" "MV: no"	In-ICU death
7	Any	"Propofol: yes" "MV: yes"	"Propofol: no" "MV: no"	"Propofol: no" "MV: no"	In-ICU death
8	Any	Any	"Propofol: no" "MV: no"	"Propofol: yes" "MV: no"	In-ICU death
9	Any	"Propofol: no" "MV: no"	"Propofol: yes" "MV: no"	"Propofol: yes" "MV: no"	In-ICU death

MV - mechanical ventilation; ICU - intensive care unit.

If it is decided to either terminate supporting therapies or, based on the presumed wishes of the patient (e.g., communicated by a surrogate) or on an advance directive, not to start or to increase life-sustaining therapies (e.g., oxygen supply), propofol may be started for sedation. Death usually occurs one to two days after these therapeutic changes. To account for this bias within the transition from the state "propofol: no" to the absorbing state "propofol: yes", we will re-classify the subsequent absorbing state "in-ICU death" into "palliative in-ICU death", if death occurs one or two days after the preceding state transition (time course of states # 8 - 9, Table 3), and into "non-palliative death" in all other time courses of states.

For the reasons described in b), the preexisting propofol dose can be increased acutely to relieve symptoms. Therefore, when analyzing transitions to the absorbing ‘in-ICU death’ state in the combination with a certain preceding propofol-associated energy intake in the initial state, we will re-define the absorbing state "in-ICU death" into "palliative in-ICU death", if there is a significant increase of propofol-associated energy intake from one day to the other ([propofol-associated energy intake on transition day minus energy intake on the day before transition day]/energy intake on the day before transition day = > 100%), or if propofol-associated energy intake on transition day is > 4.5kcal/kg day, and into "non-palliative death" with all other changes/intake.

**Figure 2:**
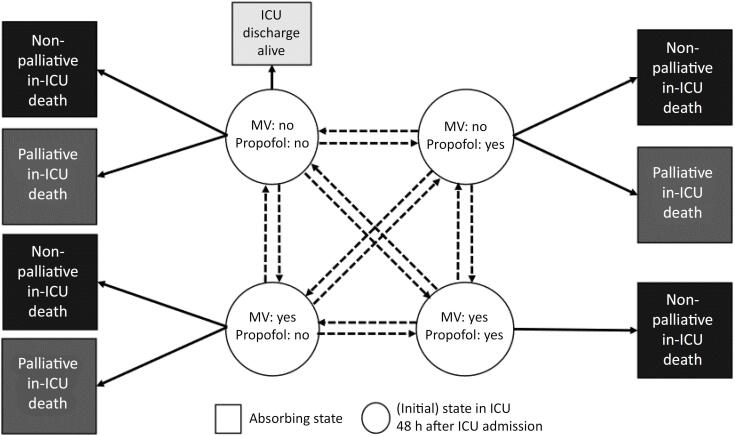
Modified multistate model describing the transitions between different clinical states depending on the risk of a palliative care bias.

Consequently, depending on each patient's history, these sensitivity analyses will increase the number of possible state transitions (Figure 2) compared to the primary analysis (Figure 1).

ICU - intensive care unit; MV - mechanical ventilation.

For details, see Figure 1. Time courses of different daily states used to re-define the absorbing state "in- intensive care unit death" into "palliative in- intensive care unit death" and "non-palliative in- intensive care unit death" are presented in Table 3.

### Sensitivity analysis for the competing risk models used to analyze time-to-discharge alive/in-hospital death

When analyzing the cumulative association between propofol-associated energy intake and these outcomes, we will use a lag time of two days to minimize indication bias due to lower doses approaching discharge (or to higher doses before death). Consequently, the time window (defined by the lead time, see above) for which cumulated associations of propofol-associated energy intake with time-to-discharge alive/in-hospital death are calculated will be shortened by two days.

### Limitations

This study is subject to the inherent limitations of observational studies, including selection bias, information bias, confounding due to informative missing data (e.g., malignant diseases), confounding by indication, and residual confounding due to unmeasured variables. In terms of propofol use, confounding by indication is highly relevant. The main confounding factor is the concurrent use of MV, which indicates underlying respiratory dysfunction. This could lead to an erroneous association between propofol use and a worse outcome, when in fact the worse outcome is caused by the respiratory dysfunction. To minimize this bias, all models will adjust for the concomitant duration of MV in propofol-related associations. Another confounding factor is the use and dosing of propofol in end-of-life care. To minimize this bias, sensitivity analyses will be performed that exclude suspicious state transitions and events occurring close to the last day of propofol therapy (lag time).

Due to the characteristics of our database, we do not have information on concomitant central nervous system dysfunction (e.g., septic *delirium*) in the absence of MV use. This type of organ dysfunction may also require propofol administration and may also be associated with a worse outcome. We have attempted to account for at least some of this bias by adjusting propofol-related associations with the outcome for baseline APACHE II scores (which contain information on the Glasgow Coma Score) and the sepsis admission diagnosis.

Another limitation is that we will be unable to analyze state transitions (multistate models) occurring within 24 hours due to missing information. Furthermore, as propofol data collection ended on day 11 after ICU admission, transition hazards can only be evaluated up to this point, and associations of propofol use with outcomes can only refer to this therapeutic period. However, as recent randomized studies (e.g., SPACE III) only recorded interventional sedation for a median of 2.6 days (IQR 1.10 - 5.3 days),^(30)^ we believe that the maximum therapeutic period (up to day 11 after ICU admission) should provide sufficient time to draw meaningful conclusions about propofol use.

Patients had not been followed after hospital discharge. Therefore, hospital discharge is treated as an endpoint, with in-hospital death as a competing event, and is accounted for using competing risks modeling (cause-specific and Fine and Gray approaches).

### Strengths

The major strength of this study is the large number of patients presenting with a wide range of diseases, enabling broad generalization of the results. Furthermore, our detailed statistical analysis plan enables us to consider two time-dependent variables (the use of propofol and MV) simultaneously, which is a common and serious limitation in observational studies.

### Ethics and dissemination

Institutional ethical approval was granted by the Health Sciences Research Ethics Board at Queen's University, Kingston, Ontario (File No: 6004791, Title: Improving the Practice of Nutrition Therapy in the Critically Ill: An International Quality Improvement Project; Initial approval: 2006/10/10).

Patients’ identities will be kept confidential and will not be disclosed or published under any circumstances, and all legal requirements for the protection of personal data will be observed. Patient data collected in the study will be documented pseudonymously using de-identified patient ID codes. All parties involved in data management and analysis will have access only to non-identifiable patient data.

In addition to this publication, details of the analysis of nutritional data (including fat intake) from the current database have been made publicly available through trial disclosure databases (ISRCTN1; ID number ISRCTN17829198).

To date, this cohort study represents the most extensive data collection of real-world evidence relevant to clinical nutrition and its impact on clinical outcomes in critically ill patients with an ICU LOS > 2 days treated in international ICUs. We therefore plan to disseminate the results at national and international conferences and submit for publication, with authorship following ICMJE recommendations (https://www.icmje.org). In addition, it is also planned that the results will be presented at local congresses and published in national journals.

## Data Availability

All data collected during the study were recorded by designated site personnel in a dedicated repository and stored in a study-specific database.

## Appendix A

Calculation of mg propofol from propofol calories in the context of a simultaneous use of propofol 1% and 2% (the ratio between those two in relation to their respective uses is assumed to be 1: 1.8):

(I)
$$\begin{array}{l} \text{Total propofol calories}\left[{\text{C}}_{\text{Pt}}(\text{kcal})\right]\text{are the sum of calories from propofol}1\text{\%}\left[{\text{C}}_{\text{P}1\text{\%}}(\text{kcal})\right]\text{and propofol}2\text{\%}\left[{\text{C}}_{\text{P}2\text{\%}}(\text{kcal})\right]\\ {\text{C}}_{\text{Pt}}(\text{kcal})={\text{C}}_{\text{P}1\text{\%}}(\text{kcal})+{\text{C}}_{\text{P}2\text{\%}}(\text{kcal}) \end{array}$$

(II)
$$\begin{array}{l} \text{Since}1\text{ml propofol contains}1.1\text{kcal, we can convert calories in ml of propofol}1\text{\%}\left[{\text{P}}_{1\text{\%}}(\text{mL})\right]\text{and propofol}2\text{\%}\left[{\text{P}}_{2\text{\%}}(\text{mL})\right]\text{:}\\ {\text{P}}_{1\text{\%}}(\text{mL})={\text{C}}_{\text{P}1\text{\%}}(\text{kcal})/1.1 \end{array}$$

(III)
$${\text{P}}_{2\text{\%}}(\text{mL})={\text{C}}_{\text{P}2\text{\%}}(\text{kcal})/1.1$$

Since 1mL of propofol may contain either 10mg or 20mg of propofol, we can calculate concentration-specific amounts of propofol [P_1%_ (mg) and P_2%_ (mg)]

(IV)
$${\text{P}}_{2\text{\%}}(\text{mg})={\text{P}}_{2\text{\%}}(\text{mL})\times 20$$

(V)
$${\text{P}}_{1\text{\%}}(\text{mg})={\text{P}}_{1\text{\%}}(\text{mL})\times 10$$

Furthermore, we know that total propofol [P_t_ (mg)] must be the sum of P_2%_ (mg) and P_1%_ (mg)

(VI)
$${\text{P}}_{2\text{\%}}(\text{mg})+P_{1\text{\%}}(\text{mg})={\text{P}}_{\text{t}}(\text{mg})$$

We also know that the ratio between P_2%_ (mg) and P_1%_ (mg) is 1.8: 1

(VII)
$${\text{P}}_{2\text{\%}}(\text{mg})/{\text{P}}_{1\text{\%}}(\text{mg})=1.8$$

From (VI) and (VII) we derive

(IIX)
$$2.8\times {\text{P}}_{1\text{\%}}(\text{mg})={\text{P}}_{\text{t}}(\text{mg})$$

Combining equations (II) to (IIX) in equation (I) yields

C_Pt_ (kcal) = [P_t_ (mg) x 1.8 × 1.1] / [2.8 × 20] + [P_t_ (mg) x 1.1] / [2.8 × 10]

Or P_t_ (mg) = 13.5 x C_Pt_ (kcal)

## References

[1] Kotani Y, Pruna A, Turi S, Borghi G, Lee TC, Zangrillo A (2023). Propofol and survival: an updated meta-analysis of randomized clinical trials. Crit Care.

[2] Shehabi Y, Serpa A, Howe BD, Bellomo R, Arabi YM, Bailey M, SPICE III Study Investigators (2021). Early sedation with dexmedetomidine in ventilated critically ill patients and heterogeneity of treatment effect in the SPICE III randomised controlled trial. Intensive Care Med.

[3] FDA issues warning on propofol (Diprivan) (2001). CMAJ.

[4] Kotani Y, Pruna A, Lee TC, Roth D, Landoni G (2023). Authors’ reply to the comment from Benavides-Zora et al. Crit Care.

[5] Ho KM, Ng JY (2008). The use of propofol for medium and long-term sedation in critically ill adult patients: a meta-analysis. Intensive Care Med.

[6] Garcia R, Salluh JI, Andrade TR, Farah D, da Silva PS, Bastos DF (2021). A systematic review and meta-analysis of propofol versus midazolam sedation in adult intensive care (ICU) patients. J Crit Care.

[7] Heybati K, Zhou F, Ali S, Deng J, Mohananey D, Villablanca P (2022). Outcomes of dexmedetomidine versus propofol sedation in critically ill adults requiring mechanical ventilation: a systematic review and meta-analysis of randomised controlled trials. Br J Anaesth.

[8] Zhang Z, Chen K, Ni H, Zhang X, Fan H (2017). Sedation of mechanically ventilated adults in intensive care unit: a network meta-analysis. Sci Rep.

[9] Hartl WH, Kopper P, Xu L, Heller L, Mironov M, Wang R (2024). Relevance of protein intake for weaning in the mechanically ventilated critically ill: analysis of a large international database. Crit Care Med.

[10] American Thoracic Society; Infectious Diseases Society of America (2005). Guidelines for the management of adults with hospital-acquired, ventilator-associated, and healthcare-associated pneumonia. Am J Respir Crit Care Med.

[11] International consensus conferences in intensive care medicine: Ventilator-associated lung injury in ARDS (1999). This official conference report was cosponsored by the American Thoracic Society, The European Society of Intensive Care Medicine, and The Societé de Réanimation de Langue Française, and was approved by the ATS Board of Directors, July 1999. Am J Respir Crit Care Med.

[12] MacIntyre NR, Cook DJ, Ely EW, Epstein SK, Fink JB, Heffner JE, American College of Chest Physicians; American Association for Respiratory Care; American College of Critical Care Medicine (2001). Evidence-based guidelines for weaning and discontinuing ventilatory support: a collective task force facilitated by the American College of Chest Physicians; the American Association for Respiratory Care; and the American College of Critical Care Medicine. Chest.

[13] Jacobi J, Fraser GL, Coursin DB, Riker RR, Fontaine D, Wittbrodt ET, Task Force of the American College of Critical Care Medicine (ACCM) of the Society of Critical Care Medicine (SCCM), American Society of Health-System Pharmacists (ASHP), American College of Chest Physicians (2002). Clinical practice guidelines for the sustained use of sedatives and analgesics in the critically ill adult. Crit Care Med.

[14] Kotani Y, Pruna A, Landoni G (2023). Mechanisms of action of the detrimental effects of propofol on survival. J Cardiothorac Vasc Anesth.

[15] Hartl WH, Kopper P, Bender A, Scheipl F, Day AG, Elke G (2022). Protein intake and outcome of critically ill patients: analysis of a large international database using piece-wise exponential additive mixed models. Crit Care.

[16] Riley RD, Snell KI, Ensor J, Burke DL, Harrell FE, Moons KG (2019). Minimum sample size for developing a multivariable prediction model: Part II - binary and time-to-event outcomes. Stat Med.

[17] Critical Care Nutrition REDCap V2 VICToRY.

[18] Charrière M, Ridley E, Hastings J, Bianchet O, Scheinkestel C, Berger MM (2017). Propofol sedation substantially increases the caloric and lipid intake in critically ill patients. Nutrition.

[19] Hastings J, Ridley EJ, Bianchet O, Roodenburg O, Levkovich B, Scheinkestel C (2018). Does propofol sedation contribute to overall energy provision in mechanically ventilated critically ill adults? A retrospective observational study. JPEN J Parenter Enteral Nutr.

[20] Matejovic M, Huet O, Dams K, Elke G, Vaquerizo Alonso C, Csomos A (2022). Medical nutrition therapy and clinical outcomes in critically ill adults: a European multinational, prospective observational cohort study (EuroPN). Crit Care.

[21] Therneau T, Crowson C, Atkinson E (2024). Multistate models and competing risks.

[22] Therneau TM, Ou FS (2024). Using multistate models with clinical trial data for a deeper understanding of complex disease processes. Clin Trials.

[23] Bender A, Groll A, Scheipl F (2018). A generalized additive model approach to time-to-event analysis. Stat Model.

[24] Bender A, Scheipl F, Hartl W, Day AG, Küchenhoff H (2019). Penalized estimation of complex, non-linear exposure-lag-response associations. Biostatistics.

[25] Kopper P, Scheipl F (2020). Flexible estimation of complex effects in the context of competing risks survival analysis: exposure-lag-response association of artificial nutrition and patients’ length of stay in intensive care units [tesis].

[26] Hartl WH, Bender A, Scheipl F, Kuppinger D, Day AG, Küchenhoff H (2019). Calorie intake and short-term survival of critically ill patients. Clin Nutr.

[27] Nießl A, Beyersmann J, Loos A (2020). Multistate modeling of clinical hold in randomized clinical trials. Pharm Stat.

[28] Dayan N, Beauchamp ME, Alcantara M, Shapiro GD, Abrahamowicz M (2025). Weighted cumulative exposure modelling to assess the association between reproductive factors and future cardiovascular disease in women. Paediatr Perinat Epidemiol.

[29] Fine JP, Gray RJ (1999). A proportional hazards model for the subdistribution of a competing risk. J Am Stat Assoc.

[30] Shehabi Y, Howe BD, Bellomo R, Arabi YM, Bailey M, Bass FE, ANZICS Clinical Trials Group and the SPICE III Investigators (2019). Early sedation with dexmedetomidine in critically ill patients. N Engl J Med.

